# Diagnosing Thoracic Outlet Syndrome: Current Approaches and Future Directions

**DOI:** 10.3390/diagnostics8010021

**Published:** 2018-03-20

**Authors:** Sebastian Povlsen, Bo Povlsen

**Affiliations:** 1 King’s College Hospital NHS Foundation Trust, Denmark Hill, London SE5 9RS, UK; 2 London Hand Clinic, London Bridge Hospital, London SE1 2PR, UK; bo@manusmedical.com

**Keywords:** thoracic outlet syndrome, neurogenic, venous, arterial, diagnosis, clinical, neurography, diffusion tensor imaging, ultrasound, dynamic CT angiography

## Abstract

The diagnosis of thoracic outlet syndrome (TOS) has long been a controversial and challenging one. Despite common presentations with pain in the neck and upper extremity, there are a host of presenting patterns that can vary within and between the subdivisions of neurogenic, venous, and arterial TOS. Furthermore, there is a plethora of differential diagnoses, from peripheral compressive neuropathies, to intrinsic shoulder pathologies, to pathologies at the cervical spine. Depending on the subdivision of TOS suspected, diagnostic investigations are currently of varying importance, necessitating high dependence on good history taking and clinical examination. Investigations may add weight to a diagnosis suspected on clinical grounds and suggest an optimal management strategy, but in this changing field new developments may alter the role that diagnostic investigations play. In this article, we set out to summarise the diagnostic approach in cases of suspected TOS, including the importance of history taking, clinical examination, and the role of investigations at present, and highlight the developments in this field with respect to all subtypes. In the future, we hope that novel diagnostics may be able to stratify patients according to the exact compressive mechanism and thereby suggest more specific treatments and interventions.

## 1. Introduction

Since its inception in 1956 by Peet et al. [1], thoracic outlet syndrome (TOS) has been used to refer to a constellation of symptoms resulting from neurovascular compression at the thoracic outlet, usually resulting in some combination of pain in the neck and upper extremity, weakness, sensory loss, paraesthesias, swelling, and discoloration [2]. The exact constellation of signs and symptoms depends on the exact structures being compressed, but the common symptoms of pain can result from either damage to the subclavian vein, subclavian artery, or different parts of the brachial plexus [3]. Classification systems have used these anatomical structures to subdivide TOS into venous TOS (VTOS), arterial TOS (ATOS), and neurogenic TOS (NTOS). Although diagnosis of any subtype of TOS can prove challenging, the diagnosis of NTOS is particularly difficult due to the branching anatomy of the brachial plexus leading to different constellations of pain, sensory disturbance, and weakness depending on the exact parts being compressed. This perhaps can be exemplified by the fact that many classification systems include a further subcategory of “disputed TOS”, where a diagnosis of NTOS is uncertain (perhaps due to the lack of supporting nerve conduction studies) but where the symptomatology is consistent with it [4]. This subdivision is losing its basis in some cases [5]. The difficulty and lack of clarity on the subject of TOS has been acknowledged by the Society for Vascular Surgery [5] from the point of view of both diagnostic criteria and a lack of evidence-based treatment after findings from a Cochrane Collaboration review [6]. Whilst the contribution of imaging and other investigations is variable in diagnosing TOS, the clinical acumen of the practicing physician or surgeon remains key to differentiating the variable presentations of TOS with the numerous potential differential diagnoses of pain in the upper extremity. Here we aim to summarise the diagnostic approach in cases of possible TOS and discuss developments in the field.

## 2. History

Good clinical acumen always begins with accurate history taking. The common theme to all presentations of TOS is the presence of pain. Questioning should focus on the precise distribution of the pain, its character, and what activities exacerbate the pain. The associated symptomatology should also be sought, as well as the resulting functional limitation experienced by the patient. A summary of findings from the history can be found in Table 1.

The distribution of pain can be very wide in NTOS, but commonly occurs in the neck, trapezius, shoulder, arm, and in some cases also manifests as chest pain and occipital headache. The pain should be non-radicular in nature and be present during activities, where it may limit function, as well as at rest. Paraesthesias can be widespread in the upper extremity and fingers. Non-specific descriptions include heaviness with movements above the shoulder, referring to the weakness of the affected muscle groups [7]. Once this is suspected, history taking may enable one to separate whether the upper or lower parts of the brachial plexus are more involved, with lower plexus (C8-T1) resulting in symptoms in the ulnar forearm and hand, as well as the axillary and anterior shoulder region. Upper plexus compression (C5-C7) results in more supraclavicular symptomatology, with radiations to the chest, periscapular region, and the head and in the distribution of the radial nerve [2]. History should also focus on potential differential diagnoses. If radicular pain is present, cervical radiculopathy should be suspected [8]. Carpal tunnel syndrome and cubital tunnel syndrome, meanwhile, should be suspected if pain and paraesthesia is confined solely to the distribution of the median and ulnar nerves distal to the point of compression in the carpal and cubital tunnels, respectively [9,10]. However, it must be remembered that these may co-exist with a diagnosis of TOS [11].

VTOS will also present with pain of the upper extremity, which may also involve the chest and shoulder. However, this is typically a “deeper” pain and one that is worse with activity. Significant associated symptoms include swelling and cyanotic discoloration of the upper extremity [12]. Sometimes, a presentation will be due to intermittent compression at the costoclavicular junction, in which case it can be aggravated or can present thrombosis of the subclavian vein (Paget–Schroetter syndrome), in which case the symptoms may be more constant and the swelling and discoloration more prominent [2].

Finally, whilst ATOS will also present with a non-radicular pain of the upper extremity, prominent features also include numbness, coolness, and pallor. Distinguishing its distribution from NTOS, pain is rarely present in the shoulder or neck [11]. Claudication may be present, but pain may also be present at rest but worsened by elevating the arms above the head. The symptoms may be intermittent initially due to compression of the subclavian artery by a cervical rib, manifesting as intermittent pain and Raynaud’s phenomenon, before complications arising from arterial damage occur (such as aneurysm, thrombosis, and critical limb ischaemia) [2].

It is becoming increasingly recognised that high-level repetitive physical activity involving the upper extremity may put individuals at risk for development of thoracic outlet syndrome. Indeed, in one centre >40% of patients requiring first rib resection and scalenectomy for NTOS relief were competitive athletes [13]. This risk also appears to extend to the vascular subtypes of TOS, where such events may be antecedents for effort-induced thrombosis [14]. Although cases of thoracic outlet syndrome in musicians have been documented [15], until now it had not been rigorously studied. A recent paper in this journal [16] prospectively evaluated 64 high-performance string instrument musicians and 52 healthy age-matched controls. They found positive elevated arm stress test (EAST) or upper limb tension test (ULTT) in 44% of musicians compared with 3% in the control group. Abnormal ultrasound scan with vascular compressions was detected in 69% of musicians versus 15% of controls. Interestingly, they also noted abnormal ultrasound scans with vascular compression were more commonly noted in violinists and viola players than cellists. Furthermore, in violinists and viola players, the left arm, which is elevated to hold up the instrument, was more commonly affected than the right bow-holding hand. This underscores the theory that it is the overhead repetitive-strain aspect of these activities that predisposes to thoracic outlet syndrome. Longitudinal studies of such patient groups would be useful to assess the likelihood of these findings progressing into clinically significant thoracic outlet syndrome requiring surgery. In the meantime, such pre-disposing factors should be ascertained in the history.

## 3. Examination

By this point in the consultation, a list of differential diagnoses including TOS may start to form. The purpose of clinical examination is to refute some and give weight to others. The general approach to a full evaluation should include a general inspection of the patient with attention to the affected limb in comparison to the contralateral limb, an examination of the cervical spine and neck including the scalene triangle, an examination of the shoulder, a full neurological examination of the upper limbs, a peripheral vascular examination, and the performance of provocative manoeuvres [5]. A summary of examination findings can be found in Table 1.

General inspection should focus on the asymmetry between the affected and contralateral limb. Signs of swelling and cyanotic discoloration may be in keeping with VTOS, whereas the observable Raynaud’s phenomenon, upper limb ischaemia, digital ulceration, and signs of peripheral embolisation may be more in keeping with ATOS [2]. NTOS on the other hand may manifest with muscular atrophy, although this is rare—pay attention to the thenar eminence, hypothenar eminence, and the interossei [17]. A clinician should also look for signs of trauma to the chest, clavicle, shoulder, and ribs, which might lead to pathological compression at the thoracic outlet [5].

Examination of the neck, cervical spine, and shoulder should include palpation of the reported sites of pain for tenderness. Palpation at possible sites of compression, such as the supraclavicular scalene triangle or subcoracoid pectoralis minor insertion site, may reproduce symptoms in NTOS [5]. If intrinsic pathology of the shoulder itself is suspected, such as subacromial impingement, adhesive capsulitis or rotator cuff injuries, a full orthopaedic examination of the shoulder should be carried out with Hawkins test positive in impingement [18], adhesive capsulitis resulting in pain in both passive and active movements in all directions [19], and rotator cuff injuries resulting in weakness of the supraspinatus, the infraspinatus, the teres minor, and the subscapularis [20]. Weakness in these respective muscle groups can be tested individually with the Jobe test in which the patient experiences weakness to restricted elevation with the patient’s arms at 90° of abduction and internally rotated, the hornblower’s test in which the patient experiences weakness in restricted external rotation of the shoulder with the arm held at 90° of abduction and the elbow flexed to 90°, and the lift-off test in which the patient experiences weakness when the dorsum of the hand is placed against the patient’s lumbar spine and is then instructed to move the hand away from the back in a perpendicular plane against resistance [21].

A peripheral neurological examination should assess the tone, power, reflexes, and sensation of the affected limb in comparison with the contralateral limb. The distribution of weakness, numbness, and paraesthesias may be variable. Suspicion of the upper brachial plexus (C5-C7) involvement is suggested by sensory disturbance of the arm and weakness and atrophy of the deltoid, biceps, and brachialis muscles. Lower plexus (C8-T1) involvement is suspected by weakness in the small muscles of the hand and weakness of wrist and finger flexion. Sensory loss may be more confined to the ulnar forearm and hand [22]. In reality, 85–90% of cases of NTOS may present with a combination of upper and lower plexus involvement [23]. A key part of the examination at this stage is to try and exclude peripheral compressive neuropathies such as carpal tunnel syndrome and cubital tunnel syndrome. Carpal tunnel syndrome will have sensory disturbance confined to the distribution of the median nerve and tinel’s and phalen’s tests may be positive, with sensitivities of 67% and 85% and specificities of 68% and 89%, respectively [24]. The sensory disturbance in cubital tunnel syndrome may be more similar to the presentation of lower plexus compression. However, elbow flexion may commonly exacerbate these symptoms in cubital tunnel syndrome [10]. It must be noted, however, that TOS may co-exist with these peripheral compressive neuropathies [11], so the positivity of these tests does not exclude a diagnosis of TOS.

Peripheral vascular examination should include palpation for a pulsatile mass in the supraclavicular and infraclavicular fossae, a sign of aneurysmal change. A bruit may also be auscultated [5]. Full status of the brachial, radial, and ulnar pulses should be recorded bilaterally. A blood pressure differential between arms of 20 mmHg may be found rarely in ATOS [25]. Whilst the above tests may not yield any findings in many cases of vascular TOS, provocative manoeuvres can be used to bring out these differences [2].

Commonly used provocative manoeuvres include EAST, ULTT, and Adson’s test [2]. In the EAST, the scalene triangle is narrowed by abducting the arms to 90° with the elbows flexed, and the shoulder externally rotated slightly to tilt the forearms backwards. In this position, repetitive opening and closing of the fist may reproduce symptoms and lead to a reduction in radial pulse volume. The ULTT assesses recreation of symptoms from stretching the brachial plexus by holding the arm outstretched with the shoulder abducted to 90°, extension and the wrist and tilting the neck away from the limb being tested. Adson’s test assesses for reproduction of symptoms or loss of radial pulse by extending the neck and rotating the head toward the symptomatic side whilst holding in deep inspiration. Provocative tests can add weight to a suspected diagnosis of TOS, but alone their utility is variable. One study found that 58% of random volunteers had at least one positive provocative test [26]. Indeed, when used alone, Adson’s test and the EAST have specificity of 76% and 30%, respectively, but when the two provocative manoeuvres are used in conjunction, diagnostic specificity can rise to 82% [27].

## 4. Investigations

Investigations play two roles in TOS: (1) to confirm or add weight to the diagnosis of arterial, venous, or NTOS and (2) to suggest the anatomical cause of compression. A summary of the role that investigations play in the diagnosis of TOS can be found in Table 2. Once a diagnosis of TOS is suspected on clinical grounds, it is important to characterise the anatomy of the thoracic outlet, particularly with respect to potential sources of compression, as this can guide management approaches, especially if surgery is to be considered. Here, CT, chest radiography, and cervical spine films may show the presence of a cervical rib or elongated C7 transverse process. MRI on the other hand can evaluate soft tissue structures that might contribute to compression, such as fibrous bands, and can exclude cervical root compression as a differential diagnosis [28].

When it comes to supporting the suspected diagnosis, the mainstays of tests in ATOS are duplex ultrasound, arteriography, haemodynamic testing (e.g., finger plethysmography) at rest, and, with provocative manoeuvres, CT angiography and MR angiography [5]. Invasive arteriography and angiography are for detecting complications of ATOS such as thrombosis, embolisation, and aneurysm. Due to the invasive nature of these investigations, they are usually employed as part of surgery planning rather than diagnosis alone. More non-invasive tests such as MR and CT angiography have been studied for their use in diagnosis outside of the context of surgical planning. One benefit is that they can be used to dynamically evaluate arterial compression with provocative manoeuvres. Whilst there has been some controversy as to the utility of provocative testing for VTOS given that moderate to severe venous compression is common in healthy subjects, arterial compression is far less common [29]. However, studies show MR angiography cannot always distinguish between physiologic and pathologic compression, and the findings cannot always be correlated with clinical symptoms [30]. Recent evidence, however, has re-asserted the utility of dynamic CT angiography with the findings that significant subclavian artery stenosis on dynamic CT angiography is correlated with thoracic outlet symptomatology. In this recent study, patients with either unilateral or bilateral symptoms underwent CT angiography whilst in a supine position, with the arms in abduction of 120° and in external rotation, with the head turned toward the less pathological or asymptomatic side. Forty percent of symptomatic outlets, compared with 5% of asymptomatic outlets, had subclavian artery stenosis ≥50% [31]. These findings in dynamic CT angiography are encouraging; however, while these investigations may lend weight toward a diagnosis of ATOS, a diagnosis cannot be made on these findings in isolation. The overall clinical picture, in the absence of detection of a thrombus, embolus, or aneurysm, therefore becomes paramount.

In diagnosing VTOS, duplex ultrasound is typically employed if thrombosis is suspected, with very high sensitivity and specificity of 78–100% and 82–100%, respectively. However, its use in cases without thrombosis is equivocal [12,32]. Even in the context of venous thrombosis, there are limitations to the use of ultrasound such as the shadow cast by the overlying clavicle over the proximal subclavian vein. In this situation, CT and MR venography can be used to demonstrate the extent of the thrombus, the degree of collateralisation, the point of compression, and the associated anatomical abnormalities [33]. If intervention is to be planned, such as catheter-directed thrombolysis or percutaneous transluminal angioplasty, contrast venography is the gold standard [34]. However, in the absence of thrombosis, imaging even with provocative manoeuvres will not be diagnostic on its own [29].

NTOS is a field where the contribution of diagnostic investigations is certainly changing. In cases of suspected TOS presumed not to be secondary to venous or arterial compression, classically electrodiagnostic studies were used to stratify cases of “true” NTOS from “disputed” NTOS—cases of similar symptomatology but lacking such conduction defects. However, given the fact that conduction defects may not be apparent at earlier stages, this distinction is being phased out [5]. Recently, however, there is suggestion that conduction deficits are present in a much larger group of patients with NTOS. Tsao et al. found that, upon testing the medial antebrachial cutaneous nerve and median motor nerve supplying the abductor pollicis brevis, T1 and C8 derived fibres commonly show conduction deficits. When electrodiagnostics combined medial antebrachial cutaneous nerve with median motor nerve testing, findings were abnormal in 89% of patients with NTOS [35]. This suggests that electrodiagnostic tests can still be used to support a diagnosis of TOS, even in its early stages.

Another testing modality suggested by the Society for Vascular Surgery is to inject the scalene and pectoralis minor muscles with local anaesthetic to check for alleviation of symptoms, the rationale being that the scalene triangle and pectoralis minor space are common sites of compression [5]. As alleviation of symptoms will only occur if the injected muscle is the source of compression, rather than a fibrous band for example, this also acts as a localisation test. Although this technique has been around for a while, having first been proposed by Gage in 1939 [36], it is still being adapted, such as the technique for the use of ultrasound guided anterior scalene and pectoralis minor blocks in high-performance overhead athletes who may often have subtle examination findings. In these patients, studying symptomatic changes while such patients exercise may be advisable [37]. Furthermore, it is only relatively recently that the degree of symptomatic and functional improvement has been fully characterised. Braun et al. [38] assessed the use of scalene blocks in individuals with symptomatology suggesting a diagnosis of thoracic outlet syndrome. Electrodiagnostics, imaging, and orthopaedic opinion were used to rule out differential diagnoses. Fingertip sensation was tested before and after the block to rule out changes secondary to sensory plexus nerve block. Compared with sternocleidomastoid injection controls, all patients with anterior scalene muscle blocks noted symptomatic and functional improvement after the blocks, with an increase in their work capacity in waist level push–pull tests by 93%, overhead bar push–pull tests by 108%, and extremity abduction stress test with repetitive hand gripping during static arm elevation by 104%. Time to fatigue and power also increased after the block. The hope is that by quantifying these parameters, diagnostically significant improvements in symptoms and function post-block can be more objectively assessed. Scalene blocks also appear to be prognostic in certain groups when it comes to predicting surgical outcome. In one study, lidocaine rather than botulinum toxin blocks were predictive of better outcomes in patients following transaxillary decompression. These were more noticeable in patients ≥40 years (14% improvement in surgical success) compared with patients <40 years (7% improvement), perhaps due to the fact that younger patients generally tend to have better surgical outcomes than older patients [39].

Increasingly it is being realised that MRI can be used not only to evaluate the anatomy of the thoracic outlet, in particular soft tissue structures that might be causing compression, but also to visualise actual compression of the brachial plexus directly. With the use of high-resolution MRI scanners with a 3.0 T magnetic field strength, MR neurography (MRN) allows nerve morphology and signal to be non-invasively visualised. This technique suppresses signal from surrounding soft tissue structures, including fat containing structures, and removes pulsation artefacts from flowing blood [40]. In this way, the exact site of compression as well as the structure causing the compression can be identified directly, instead of having to indirectly infer that the presence of a fibrous band may be the cause of compression in a patient with documented NTOS. Baumer et al. used high-resolution MRN in patients with suspicion of neurogenic or non-specified TOS to identify cases brachial plexus compression. All cases identified were subsequently verified by surgical exploration, showing good positive predictive value of this investigation. This study, however, did not verify by surgical exploration those without discernible defects on MRN, so the negative predictive value of this test is not known [41].

MRI can be applied in another modality: that of diffusion tensor imaging (DTI). This technique works on the principle of the non-random and distinct movement of water molecules through highly organised cell structures of myelinated nerve bundles [40]. Evidence suggests that the quantitative parameters generated with DTI (fractional anisotropy and axial, radial, and mean diffusivity) may correlate with the mechanism of neuropathy. One study found that, by correlating DTI findings with electrophysiology in an assessment of the median nerve in the carpal tunnel, axial diffusivity reflected axon integrity, whereas radial diffusivity and functional anisotropy reflected myelin sheath integrity [42]. Indeed, this technique has shown promise in other peripheral neuropathies including carpal tunnel syndrome. One study performed DTI on the median nerve in subjects with carpal tunnel syndrome and compared with control subjects and showed that the measured parameters in DTI show a highly significant difference (*p* < 0.0001) [43]. The reproducibility of findings, as well as the intra- and inter-evaluator agreement when it comes to the application of DTI to the brachia plexus, however, does have good evidence [44], with one study estimating reproducibility of 81–92% when healthy volunteers were imaged [45]. Although application of this technology to NTOS has not yet been reflected in the literature, there is evidence for the use of DTI in other brachial plexus injuries. One feasibility study aimed to use DTI at 1.5 T to detect nerve root avulsions in patients with brachial plexus injuries and found it to have an overall accuracy of 94.5%, including detection of both complete and partial avulsions [46].

Ultrasound imaging, a staple in VTOS, may also be applied to NTOS, with the benefit over MRI being the low cost and more readily available nature of the technology. Leonhard et al. demonstrated that there is a significant increase in the incidence of symptoms of NTOS in patients with brachial plexus variants in which portions of the proximal plexus pierce the anterior scalene and thereby may be susceptible to impingement within the muscle belly. These branching variants can be identified by ultrasonography. Of 22 subjects, 21% demonstrated this atypical branching anatomy on ultrasonography. Fifty percent of these subjects reported symptoms consistent with NTOS, versus 14% in those with classical brachial plexus anatomy [47]. Knowledge of the branching anatomy of the brachial plexus can be used to guide management, including surgery and may be used in future trials that stratify treatment approaches based on the branching anatomy. It has also been suggested that ultrasonography of the thoracic outlet can be used to dynamically evaluate brachial plexus compression, which would be highly suitable in the outpatient setting. In this technique, when an ultrasound probe is placed in the supraclavicular fossa, the brachial plexus can be visualised generally above and just posterior to the subclavian artery, and can be seen in relation to the surrounding anterior and middle scalene muscles. When the patient is asked to abduct the arm in performance of the EAST, reduction in the interscalene interval, compression of the brachial plexus, or obliteration of the visualised nerves can be correlated with the reproduction of symptoms to add weight to a diagnosis of NTOS [48]. This technique, however, requires more rigorous evaluation.

## 5. Summary and Perspective

As we can see, diagnosis of TOS is complicated by the variety of presentations, the number of differential diagnoses, including co-existence of diagnoses such as arterial with NTOS and peripheral compressive neuropathies with TOS, and the variable reliability of provocative manoeuvres and investigations when used in isolation. In this context, the diagnosis requires high-quality clinical acumen with respect to history taking and examination, with investigations often used more to add weight to a suspected diagnosis. As discussed, imaging is used to evaluate the anatomy of the thoracic outlet to guide management, such as whether to excise a cervical rib. However, in the absence of such abnormalities, the exact mechanism of compression may not be fully understood. It is therefore encouraging to see ongoing developments in this area, including dynamic CT angiography, MR neurography, DTI, and the use of ultrasound, including an article recently published in this journal, which showed that it can be used to identify brachial plexus branching variants in which susceptibility to compression by the scalene muscle is increased [47]. Developments of similar tools may begin to stratify patients according to the pathophysiology of compression, rather than purely on their respective clusters of symptoms, may pave the way in developing more specific treatments.

## Figures and Tables

**Table 1:** History and examination features in ATOS, VTOS, and NTOS.

TOS Subtype	History	Examination
**ATOS**	Claudication/rest pain of upper limb, excluding shoulder/neck Numbness, coolness, pallor	Raynaud’s phenomenon Upper limb ischaemia, digital ulceration, peripheral embolisation Pulsatile mass ± bruit on auscultation Blood pressure differential >20 mmHg Positive EAST, ULTT, Adson’s test
**VTOS**	Deep pain on movement or rest pain in upper limb, chest, shoulder Swelling and cyanotic discoloration	Upper limb swelling Cyanosis Positive EAST, ULTT, Adson’s test
**NTOS**	Pain in neck, trapezius, shoulder, arm, chest, occipital headache Variable pattern upper limb weakness, numbness, paraesthesias	Tenderness on palpation: scalene triangle, subcoracoid space Upper plexus (C5-C7): sensory disturbance of arm. Weakness/atrophy of deltoid, biceps, brachialis Lower plexus (C8-T1): sensory disturbance ulnar forearm & hand. Weakness/atrophy of small muscles of the hand, weak wrist & finger flexion Positive EAST, ULTT, Adson’s test

**Table 2:** Investigations in ATOS, VTOS, and NTOS.

TOS Subtype	Definite Role	Possible Role	Emerging Role
**All**	Plain radiography (chest/cervical spine) Non-contrast CT/MRI [5,27]	-	-
**ATOS**	Duplex ultrasound Contrast arteriography Finger plethysmography [5]	CT/MR arteriography with provocative manoeuvres [5,26,28,29,30]	-
**VTOS**	Duplex ultrasound [5,12,31] CT/MR venography [32] Contrast venography [33]	CT/MR venography with provocative manoeuvres [5,26,28]	-
**NTOS**	Nerve conduction studies Needle electromyography [5,34] Local anaesthetic injection test [5,35,36,37,38]	-	MR neurography [39,40] Diffusion tensor imaging [41,42,43,44,45] Brachial plexus ultrasound [46,47]
